# Identification of diagnostic DNA methylation markers in the blood of Japanese Alzheimer’s disease patients using methylation capture sequencing

**DOI:** 10.1186/s13148-025-01905-0

**Published:** 2025-06-20

**Authors:** Risa Mitsumori, Kayoko Sawamura, Kimi Yamakoshi, Akinori Nakamura, Yutaka Arahata, Shumpei Niida, Daichi Shigemizu, Kouichi Ozaki, Nobuyoshi Shimoda

**Affiliations:** 1 Medical Genome Center, National Center for Geriatrics and Gerontology, Research Institute, Obu, Aichi Japan; 2 Center for Development of Advanced Medicine for Dementia, National Center for Geriatrics and Gerontology, Obu, Aichi Japan; 3 National Center for Geriatrics and Gerontology, National Hospital for Geriatric Medicine, Obu, Aichi Japan; 4 National Center for Geriatrics and Gerontology, Research Institute, Obu, Aichi Japan; 5 Department of Cardiovascular Medicine, Hiroshima University Graduate School of Biomedical and Health Sciences, Hiroshima, Japan; 6 RIKEN Center for Integrative Medical Sciences, Yokohama, Japan

## Abstract

**Background:**

Methylation capture sequencing (MC-seq), which relies on next-generation sequencing technology, offers advantages over the widely used array-based approach that Illumina Inc. developed regarding both resolution and comprehensiveness for detecting DNA methylation changes across genomes. In the present study, MC-seq was employed for the first time to identify DNA methylation markers for Alzheimer’s disease (AD).

**Results:**

We compared DNA methylation in the blood of 12 AD patients with brain amyloidosis and 12 cognitively normal elderly Japanese individuals without brain amyloidosis. Candidate methylation differences were validated in the two cohorts using bisulfite amplicon sequencing. Significant differentially methylated regions were identified in the *ANKH*, *MARS*, *ANKFY1*, *LINC00908*, and *KLF2* genes and a slight methylation change in *CHRNE* (*p* = 0.061). Furthermore, our AD diagnostic prediction model showed that combining the methylation levels of *ANKH* and *MARS* with the *APOE* genotype provided diagnostic accuracy, achieving AUCs of 0.90 and 0.81 in the discovery and validation datasets, respectively.

**Conclusions:**

The present results suggest the potential of combining these markers for diagnosing AD and support the validity of our approach for identifying disease-related DNA methylation markers using next-generation sequencing.

**Supplementary Information:**

The online version contains supplementary material available at 10.1186/s13148-025-01905-0.

## Background

In vertebrate genomes, cytosine bases at cytosine–phosphate–guanine dinucleotide sites (CpGs) are the primary substrates for methylation by DNA methyltransferases. Due to its stability in blood samples [1], susceptibility to environmental factors, and regulatory roles in gene expression, DNA methylation is a promising biomarker and a potential key to identifying genes associated with complex diseases, including Alzheimer’s disease (AD). Many CpG sites associated with AD have been identified by comprehensive microarray DNA methylation analyses and targeted gene approaches using blood- and brain-derived DNA [2]. However, few CpGs have been replicated across independent studies [2]. This lack of reproductivity may be attributed to unresolved limitations in microarray methylation analyses [3–5]. Furthermore, microarrays capture only 3% of the 28 million CpGs in the human genome, suggesting that many AD-associated CpGs have yet to be discovered. The identification of hidden CpGs in blood samples will contribute to the more accurate prognostication and diagnosis of AD as well as a more detailed understanding of its etiology.

To discover novel and reproducible DNA methylation changes associated with AD, we employed methylation capture sequencing (MC-seq), also known as targeted bisulfite sequencing, an alternative method originally named solution hybrid selection bisulfite sequencing [6]. Using next-generation sequencing (NGS), MC-seq directly quantifies methylation levels at individual CpG sites in DNA fragments captured by biotinylated RNA or DNA probes designed for specific genomic regions. In this study, we selected the TruSeq Methyl Capture EPIC library prep (TruSeq EPIC) from Illumina Inc., which enables methylation quantification at more than 3.3 million CpG sites per individual, representing 12% of all CpGs in the human genome. Capture probes target 107 Mb of genomic DNA (gDNA), covering key regulatory elements, such as promoters, enhancers, CpG islands, and CpG island shores/selves [7]. MC-seq has already successfully identified a number of disease-related markers [8–10]; however, to the best of our knowledge, it has not yet been applied to AD research. Although MC-seq is more cost effective than whole-genome bisulfite sequencing [11], the processing of large sample sets remains expensive. Therefore, in the present study, we used MC-seq to identify differentially methylated regions (DMRs) in the first cohort composed of 12 AD patients (Pittsburgh compound-B positron emission tomography (PiB-PET)-positive) and 12 matched cognitively normal (CN) elderly individuals (PiB-PET-negative). Candidate DMRs detected through MC-seq were validated in two independent cohorts of AD and CN individuals using whole-blood DNA and bisulfite amplicon sequencing (BA-seq), another NGS-based method [12].

## Methods

### Subjects

The cognitive status and dementia severity of subjects were assessed using the Mini-Mental State Examination (MMSE) by trained physicians at the National Center for Geriatrics and Gerontology (NCGG) hospital. The first cohort was composed of 12 AD cases and 12 CN elderly individuals, with and without brain amyloidosis, as detected by PiB-PET scanning at the NCGG hospital. In a replication analysis, we prepared second and third cohorts, each consisting of 48 clinically diagnosed AD cases and 48 CN individuals, all aged 60 years or older. Clinical information, including MMSE scores, sex, age, and the apolipoprotein E (*APOE*) epsilon (ε) allele genotype, was obtained from the NCGG biobank.

### Preparation of gDNA

gDNA was extracted from peripheral blood using a Maxwell RSC Instrument (Promega, USA) with the Maxwell RSC Buffy Coat DNA Kit (Promega, USA). The quantity of gDNA was measured using the Quant-iT 1 × dsDNA BR Assay (Thermo Fisher Scientific, USA), and the intactness of gDNA was assessed by electrophoresis on 1.0% agarose gels.

### Sodium bisulfite treatment of DNA

In a methylation analysis, 200 ng of DNA samples was treated with sodium bisulfite to convert methylated cytosine to cytosine and demethylated cytosine to uracil, either by the EZ DNA Methylation-Gold Kit or EZ-96 DNA Methylation-Gold Kit (Zymo Research, USA) according to the manufacturer’s instructions.

### MC-seq

We processed twenty-four samples of gDNA from the first cohort using the target enrichment system, the TruSeq methyl capture EPIC Kit (Illumina, Inc., CA, USA). Captured DNA fragments were sequenced by NGS as follows: gDNA was fragmented to a few hundred base pairs (bps) by a sonicator (M220, Covaris), followed by blunting, phosphorylation, the addition of 3’-dA, and the ligation of indexed adaptors to both ends of DNA. DNA was mixed and hybridized to capture oligos, and streptavidin-conjugated magnetic beads recovered hybridized DNA. Eluted DNA from magnetic beads was treated with sodium bisulfite and amplified by PCR to construct a sequencing library, which was sequenced using the Illumina HiSeq 2500 platform with paired-end reads of 100 bp according to the manufacturer’s instructions. Library construction and sequencing were conducted by Takara Bio, Inc. (Shiga, Japan). Raw sequence data were converted from a bcl file to a fastq file using Illumina bcl2fastq2 Conversion software v2.17. Sequencing data were aligned to the reference sequence (Genome Reference Consortium Human Build 37 (GRCh37)/UCSCgh19) using the application, MethylSeq, through the cloud service named BaseSpace (Illumina, Inc., CA, USA). The numbers of methylated and unmethylated cytosines at each CpG site, namely the methylation call, were counted by MethylKit, another application available through BaseSpace. The same application used methylation call data to detect differential DNA methylation between AD subjects and CN elderly. We set the conditions of MethylKit for differentially methylated CpG sites to be identified as follows: ≥ 10 coverage with independent reads and ≥ 15% methylation differences with a *q* value ≤ 0.01.

### Definition of DMR

**Fig. 1:**
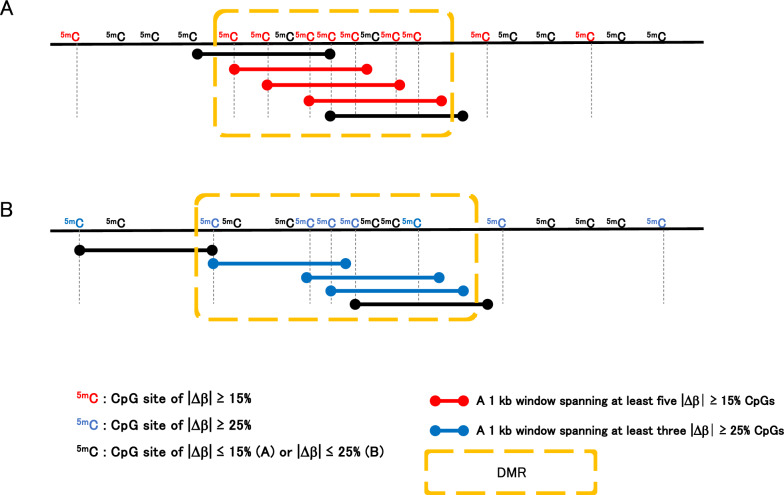
An image of our sliding window approach to define DMRs. In this study, CpGs that meet either of the following two conditions are defined as constituting a DMR: at least five or three CpG sites of |*Δ*β|≥ 15% (A) or |*Δ*β|≥ 25% (B) in 1 kb, respectively. We regard consecutive DMRs, as shown above, as one DMR. *Δ*β is the average methylation level (%) difference at the same CpG site between the CN and AD genomes

Genomic regions where differentially methylated cytosines cluster are referred to as DMRs, although the criteria for defining DMR may be arbitrary. In the first cohort, we identified DMRs using a sliding window approach, as shown in Fig. 1. We initially searched for genomic regions containing at least five or three cytosines with consistent methylation differences (|*Δ*β|≥ 0.15 or |*Δ*β|≥ 0.25, respectively) within a 1-kb window. The window was then slid up to 1 kb in either or both directions as long as the new window contained a combination of differentially methylated cytosines that met the above criteria. A region was defined as a DMR if even a single window met these conditions.

Our definition of the DMR threshold was arbitrary and based on a practical reason: Since there is no precedent to obtain AD-related methylation markers by MC-seq and the sample size of the first cohort was small, there is currently no information available on how to set a reasonable threshold to define a DMR. However, prior to the initiation of the present study, we decided to use an Illumina sequencing kit to simultaneously process 96 amplicons for the validation experiment, and we also aimed to limit the number of clones to 96 for BA-seq as a labor- and time-saving strategy. A total of 106 DMRs remained by setting |*Δ*β|≥ 0.15 on MethylKit based on an exploratory analysis; therefore, we defined |*Δ*β|= 0.15 for the DMR threshold.

### PCR primers

PCR primers for bisulfite-treated gDNA were designed by MethPrimer [13] or by Pyrosequencing Assay Design Software ver. 2.0 provided by Qiagen (CA, USA) for pyrosequencing.

### Preparation of amplicons from the second cohort

One or multiple regions of each of the 88 DMR candidates were amplified from each subject’s DNA in the second cohort by using KOD Multi & Epi (Toyobo, Inc., Shiga, Japan) in 20 μl of the reaction mixture with the following touchdown PCR conditions: at 94 °C for 2 min; 4 cycles at 98 °C for 10 s, 64 °C for 30 s, and 68 °C for 15 s; 4 cycles at 98 °C for 10 s, 60 °C for 30 s, and 68 °C for 15 s; 4 cycles at 98 °C for 10 s, 58 °C for 30 s, and 68 °C for 15 s; and 30 cycles at 98 °C for 10 s, 55 °C for 30 s, and 68 °C for 15 s. The average length of amplicons was 237 bp. All amplicons were electrophoresed on 2% agarose gels with 0.5 × TAE buffer, followed by staining with GelRed (Biotium, USA), and PCR was repeated for unsuccessful amplification. We collected 1 μl from each of the 133 amplicons from single subjects and mixed the aliquots in single tubes. Pooled PCR products were then purified by QIAvac 96 (QIAGEN, CA, USA), which removes low-molecular weight DNA (≤ 80 bp). We electrophoresed 1 μl of each eluate on a 2% agarose gel to check the recovery of pooled PCR products. The concentrations of purified pools of amplicons were measured using a 2200 TapeStation Instrument (Agilent Technologies, Santa Clara, CA, USA).

### Preparation of amplicons from the third cohort

One or multiple regions of each of the six DMRs identified in the present study were amplified from each subject in the third cohort, and all amplicons were assessed using the method described above. Based on the amplification level judged from agarose gel electrophoresis of each amplicon, we took either 1 or 4 μl from each amplicon per subject and pooled the aliquots into single tubes. The final volume of each pooled sample was adjusted to 100 μl with distilled water. Purification of the mixture, followed by agarose gel electrophoresis, and quantification of the purified mixture were performed as described above.

### BA-seq

We constructed libraries for BA-seq from 80 ng of purified amplicon mixtures by using TruSeq Nano DNA Library Prep Kits (Illumina, USA) following the manufacturer’s instructions, except for the enrichment process of DNA fragments. We used AMPure XP (Beckman Coulter, USA) instead of the sample purification beads included in the kit. We started from the middle of the instructions because the fragmentation of DNA was unnecessary for PCR amplicons. We started with the reaction of “A-tailing,” which adds adenine to the ends of amplicons. The molarity of the libraries and the degree of the undesirable inclusion of adaptor dimers in the libraries, which appeared around 150 bp, were assessed using the Agilent D1000 screen tapes system on a 2200 TapeStation system (Agilent, USA). A 4 nM pool of libraries and 4 nM PhiX Control v3 (Illumina, USA) were each denatured for 5 min with freshly prepared 0.2 N NaOH and adjusted at concentrations of 6 and 8 pM, respectively. Denatured pools were mixed with denatured Phi X at volume ratios of 85:15 and 70:30 for the second and third cohort DNA amplicon libraries, respectively, and 600 μl of each of the prepared samples was loaded into the reagent cartridge of the MiSeq Reagent Kit v2 (Illumina, USA). The cartridge sets on the Illumina MiSeq platform were used to sequence libraries with 151 paired-end, dual-indexing cycles per read (2 × 151). The densities of clusters generated on a flow cell were 322 ± 8 and 343 ± 10 k/mm^2^ for BA-seq of the second and third cohorts, respectively.

### Data analysis of BA-Seq

We trimmed adaptor sequences from read sequences using Trim Galore (version 0.6.6), setting the trimming cutoff at 20 (-q) and using the rrbs option (–rrbs). After trimming, sequencing data were mapped to the human reference genome (GRCh37) using Bismark (version 0.22.3) with the “genome_preparation” function, an aligner optimized for bisulfite sequence data and methylation calling. The BAM files generated were sorted by chromosomes using SAMTools (“sort”). Sorted BAM files were then utilized for methylation calling and the calculation of methylation rates using the MethylKit package in R (genomeBismarkAln), with the following settings: human genome assembly “hg19,” read context “CpG,” and a minimum coverage cutoff of “10.” By using the DNA methylation rates (meC/meC + C) of CpGs in DMRs as explanatory valuables, the significance of methylation differences at DMRs was evaluated using the burden test (SKAT in R), adjusting for sex and age (kernel = “liner.weighted”).

### Selection of best DMR sets

We selected CpG sites with methylation differences between CN and AD that were significant (*P*_*bon*_ $\le$ 0.05) in the second and third cohort analyses and that showed the same direction of methylation change. Second and third cohort subjects were mixed and randomly split: Four-fifths were used for a discovery dataset and one-fifth for a validation dataset, using sklearn (“train_test_split”) in Python3. Using discovery data, prediction models were constructed based on clinical information (age, sex, and *APOE* ε_4_ genotypes), and all possible combinations of DMRs using a logistic regression model. The optimal model, incorporating clinical information and DMRs, in the discovery dataset was selected using a threefold cross-validation. After finalizing the model on the entire discovery dataset, its performance was evaluated on an independent validation dataset, with the area under the receiver operator characteristic (ROC) curve (AUC) being used to assess discriminative accuracy. Analyses were implemented using the sklearn library in Python (penalty: “elasticnet,” solver: “saga,” 1l_ratio: 0.5). Sensitivity to the values of the regularization parameter (C parameter (0.1, 01, 10, 100)) was examined with the help of simulating by the best model.

## Results

### Detection of candidate DMRs in the first cohort

The present study focused on DMRs, clusters of neighboring CpGs with significant methylation changes in the same direction. There were two main reasons for this approach. Cytosine-to-thymine transitions at CpG sites, the most frequent de novo mutation occurring in the human genome [14], are indistinguishable from unmethylated cytosines after a bisulfite treatment, potentially leading to false differentially methylated positions (DMPs). To prevent this, we considered only DMPs aggregated in a short distance, defining them as DMRs for use as diagnostic markers. Furthermore, the utility of DMRs in blood DNA as diagnostic markers for AD has already been demonstrated [15, 16].

**Fig. 2:**
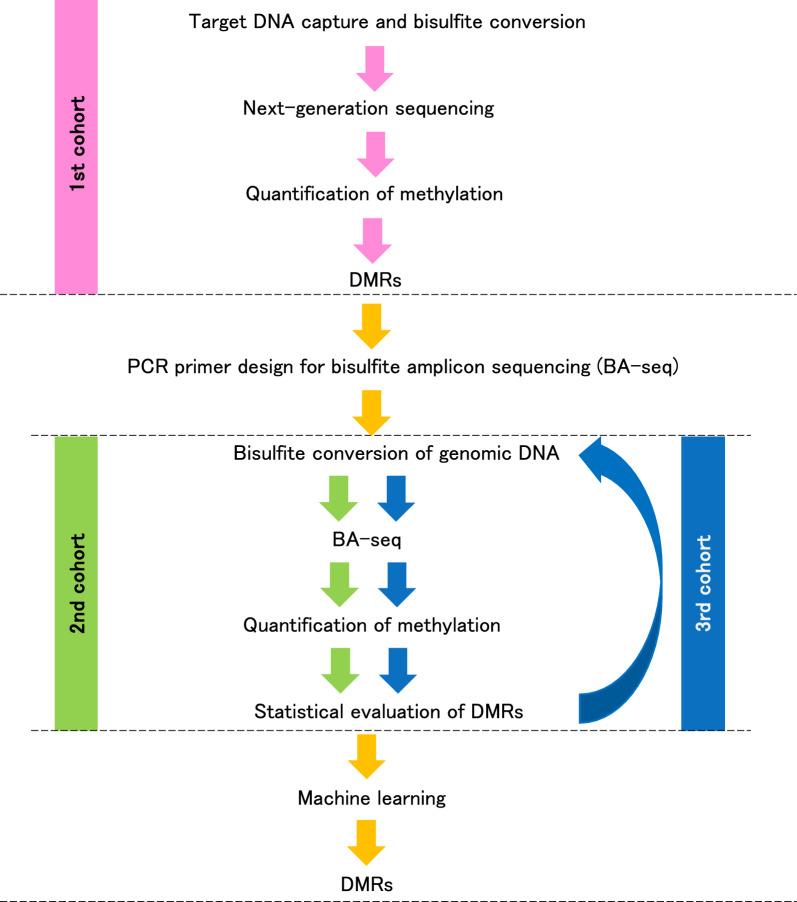
Flowchart of analysis step. Small-scale discovery analysis of the first cohort followed by replication in two independent cohorts

Our approach to comprehensively identify DMRs in the blood DNA of AD patients is shown in Fig. 2.

We first directly compared sequences from the first cohort, composed of 12 CN and 12 AD individuals, using the commercially available TruSeq Methyl Capture EPIC kit (Illumina Inc.). All donors in this cohort underwent PiB-PET imaging, with 12 CN and 12 AD individuals testing negative and positive for brain amyloidosis, respectively (Table 1).

**Table 1:** Sample characteristics of three cohorts used in this study

	1st cohort		2nd cohort		3rd cohort	
Characteristics	CN-	AD +	CN	AD	CN	AD
Samples	12	12	48	48	48	48
Male	4 (33.3)	2 (16.7)	24 (50)	24 (50)	24 (50)	24 (50)
Age, years	73.3 ± 3.2	74.4 ± 6.4	70.3 ± 2.4	71.2 ± 4.0	69.8 ± 3.0	71.8 ± 2.6
MMSE score	29.0 ± 1.6	21 ± 3.4	28.8 ± 1.3	16.2 ± 3.5	29.5 ± 0.7	16.9 ± 4.9
*APOE* $\varepsilon$ 4 homozygote	0 (0)	2 (16.7)	1 (2.1)	5 (10.4)	1 (2.1)	6 (12.5)
*APOE* $\varepsilon$ 4 heterozygote	1 (8.3)	8 (66.7)	5 (10.4)	30 (62.5)	10 (20.8)	19 (39.6)
*APOE* $\varepsilon$ 4 non-carriers	11 (91.7)	2 (16.7)	42 (87.5)	13 (27.1)	37 (77.1)	23 (47.9)
PiB-PET	negative	positive	-	-	-	-

Age and MMSE are expressed as the mean ± SD, while the other variables are expressed as numbers, n (%). Abbreviations: SD, standard deviation; MMSE, Mini-Mental State Examination; *APOE*, apolipoprotein E; -, not executed

A total of 437,792 target DNA fragments were recovered from the first cohort, yielding 107,403,887 bp of sequence data. These fragments were mapped to UCSDhg19 using Bowtie2 [17], with an average mapping rate of 83.9% (CN: 84.07 ± 0.45%; AD: 83.81% ± 0.37). The methylation rate at each CpG site was calculated by Bismark [18], which identifies methylated and unmethylated cytosines in sequenced reads. Illumina integrates both programs into the online application, MethylSeq, available through BaseSpace Sequence Hub, which was used in the present study. A total of 3,233,016 CpG sites were analyzed, covering 96.8% of the 3,340,894 CpGs detectable by the TruSeq Methyl Capture EPIC kit. These results indicated the successful construction of an enrichment library and sequencing (Table 2). An aggregated summary and raw data of MC-seq are shown in Table 3 and supplementary Table S1, respectively.

**Table 2:** Expected and actual recovery of target regions and CpGs from the first cohort

	Illumina TruSeq Methyl Capture EPIC		Template
	Expected	Actual	The human genome
Total length of targets (Mb)	107	107	3,000
Number of target regions (%)	437,792 (100%)	436,268 (99.7%^a^)	
Number of CpGs	3,340,892 (11.8%^c^)	3,233,016 (96.8%^b^)	2,8217,009 (100%)

The ratio of recovered targets^a^ or CpGs^b^ to the expected number of targets or CpGs to be recovered from the experimental design. The ratio of the expected number of CpGs to be analyzed^c^ to the total number of CpGs in the human genome

**Table 3:** Statistics of methyl capture sequencing of the first cohort

	CN-	AD +
Total PF reads^a^	135,879,157 ± 11,735,593	135,600,493 ± 22,796,635
Aligned reads^b^, %	84.07 ± 0.45	83.81 ± 0.37
Duplicate aligned reads^c^, %	12.42 ± 3.90	10.49 ± 1.70
Unique read enrichment^d^, %	79.84 ± 0.76	79.17 ± 1.87
Target coverage at least 20 × , %	93.60 ± 1.96	93.56 ± 3.12
Cytosine methylation in the CpG context, %	52.66 ± 0.69	51.91 ± 0.62
Cytosine methylation in the non-CpG context, %	1.10 ± 0.02	0.98 ± 0.03

CN- and AD + indicate cognitively normal elderly negative for amyloid PET and clinically diagnosed AD cases positive for amyloid PET, respectively. ^a^The total number of pass-filter reads for the sample. ^b^The percentage of pass-filter reads that aligned to the reference genome. ^c^The percentage of paired-end reads flagged as duplicates. ^d^100 × (Targeted unique aligned reads/Unique aligned reads). Variables are expressed as the mean ± SD

We used MethylKit [19], accessible through BaseSpace Sequence Hub, to identify DMPs that may constitute DMRs under the following stringent settings: (1) CpGs with a minimum coverage of 10 reads (coverage ≥ 10), (2) CpGs showing a methylation difference of at least 15% (|*Δ*β|≥ 15%) between CN and AD, and (3) CpGs with a *q* value < 0.01. We obtained 10,381 DMPs, including 1,077 DMPs with |*Δ*β|≥ 25%. After excluding 124 and 1 DMPs from the X and Y chromosomes, respectively, we retained 10,256 DMPs of |*Δ*β|≥ 15%, of which 1,065 had |*Δ*β|≥ 25%.

**Fig. 3:**
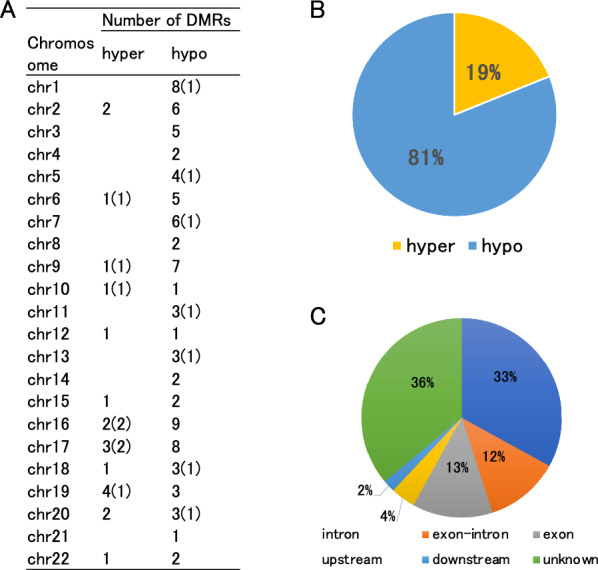
Characteristics of DMRs. MC-seq of the first cohort showed the unbiased chromosomal distribution of DMRs. (A) Chromosomal distribution of DMRs showing methylation differences ≥ 15%. Numbers in parentheses are those ≥ 25%. (B) Hypo- and hypermethylation ratios of DMRs. (C) Locations of DMRs relative to genes

To detect DMRs, we applied a sliding window analysis (Fig. 1), defining DMRs as regions containing at least seven DMPs with |*Δ*β|≥ 15% or five DMPs with |*Δ*β|≥ 25% within a genomic region of a few kb. We identified 106 DMRs, including 6 DMRs composed entirely of |*Δ*β|≥ 25% DMPs (supplementary Table S2). Of these, 86 DMRs (81%) were hypomethylated (CN > AD), while 20 (19%) were hypermethylated (CN < AD) (Fig. 3). The predominance of hypomethylation in AD blood samples is consistent with previous findings [20]. Detailed information on the positions and lengths of DMRs is shown in Supplementary Table S2.

### Validation of DMRs with the second cohort

To validate methylation differences in the 106 AD-related DMRs detected from the first cohort, we analyzed a second cohort of 48 AD cases and 48 CN individuals (none of whom underwent PiB-PET imaging, Table 1) by BA-seq. Primer sets were successfully designed for 91 DMRs, while the remaining 15 DMRs could not be targeted (Supplementary Table S3).

Primer specificity was initially examined to establish whether they uniquely PCR amplified DNA of the expected sizes from control bisulfite-treated human gDNA by electrophoresis of PCR products on 2% agarose gels. Alternative primer sets were tested for unsuccessful amplifications; however, the expected PCR products were not obtained for 3 DMRs (Supplementary Table S3). In total, 133 amplicons were ultimately generated against 88 DMRs (Supplementary Table S4), and a bisulfite amplicon DNA library was constructed for paired-end sequencing.

The genomic locations of the DMRs, their relative positions within genes, primer sequences, amplicon lengths, and other details are shown in Supplementary Table S4. Since bisulfite-treated DNA and PCR amplicons significantly reduce library diversity, we spiked the sample with 15% PhiX control and reduced the library concentration to achieve a lower cluster density on a flow cell than recommended for standard DNA sequencing. The MiSeq sequencing run yielded a cluster density of 322 ± 8 (K/mm^2^), with 82.12 ± 2.96 (%) of the cluster passing filter, and a ≥ Q30 score of 91.0%. The average read numbers per sample were similar between the CN and AD groups: 38,977 and 38,656, respectively (Table 4).

**Table 4:** Averaged results of bisulfite amplicon sequencing

	2nd cohort		3rd cohort	
Characteristics	CN	AD	CN	AD
Total number of pair-end reads	38,977 ± 4111.3	38,656 ± 4994.5	29,702 ± 5871.4	32,623 ± 8886.1
Reads uniquely mapped to the reference genome	30,747 ± 3454.8	29,541 ± 3986.6	28,341 ± 5645.9	31,103 ± 8486.8
Reads unable to be mapped at any loci	5764 ± 913.4	6501 ± 954.2	1361 ± 285.4	1519 ± 456.6
Reads mapped at multiple loci	2466 ± 377.2	2614 ± 510.4	0.25 ± 0.56	0.27 ± 0.54
Mapping efficiency (%)	78.9 ± 2.03	76.4 ± 1.70	95.4 ± 0.64	95.3 ± 0.78

All continuous variables were expressed as the mean ± SD

The average mapping rates of reads from CN and AD samples were 78.9 and 76.4%, respectively (Table 4). Among the 88 DMRs analyzed, eight amplicons corresponding to eight DMRs were excluded due to insufficient read coverage (< 10 reads) in at least one sample. Additionally, 10 amplicons representing 10 DMRs were excluded because they could not be uniquely mapped to the reference genome, likely due to the short length and low complexity of DMR sequences (Supplementary Table S5).

**Fig. 4:**
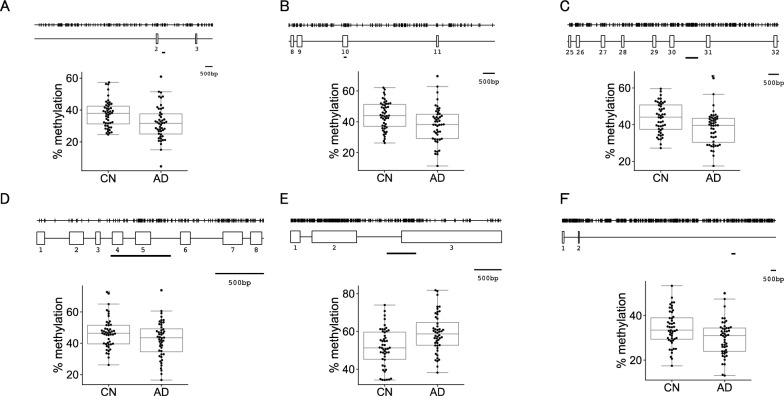
Differences in DNA methylation levels in 6 DMRs in the second cohort. The schematics depict the distributions of CpG dinucleotides along the five genes and a long intergenic non-coding (*LINC*) RNA in which DMRs were found. Vertical lines show the positions of CpG dinucleotides. Open rectangles indicate axons. Underlined were the regions for which the methylation levels were analyzed by bisulfite amplicon sequencing. The five genes and a *LINC* harboring a DMR were as follows: *ANKH* (A), *MARS* (B), *ANKFY1* (C), *CHRNE* (D), *KLF2* (E), and *LINC00908* (F)

Regarding the remaining 70 DMRs, 114 amplicons, including 1,251 CpG sites, were analyzed. Methylation differences at 511 CpGs in 67 DMRs were significant (*P*_*bon*_ ≤ 0.05/1251). Of the 67 DMRs, 52 contained 496 CpGs with at least three CpGs per DMR showing significant methylation differences. Using the DNA methylation rates (meC/(meC + C)) of these CpGs as explanatory valuables, the significance of methylation differences at these DMRs was evaluated using the burden test, adjusted for sex and age [21]. The burden test is a collapsing method used for genetic association analyses of rare variants. The method collapses variants within a gene and analyzes them as a unit to assess whether the gene is associated with a disease. In the present study, the methylation rates of CpGs in a DMR were treated as the allele frequencies of genetic variants in a gene, and the relationship with AD was tested. The *p* values of DMRs calculated by the burden test were converted to *q* values using the Benjamini–Hochberg procedure [22], and 6 DMRs were found to be significant with *q* values < 0.05 (Table 5). Five of the 6 DMRs were located in the gene body regions of the following genes: *ANKylosis Homolog* (*ANKH*), *MARS*, *ANKFY1*, *Cholinergic Receptor Nicotinic Epsilon Subunit* (*CHRNE*), and *KLF2*, while the remaining DMR was found in the long non-coding RNA gene, *LINC00908*. Of these, five DMRs in *ANKH*, *MARS*, *ANKFY1*, *CHRNE*, and *LINC00908* showed lower methylation levels in AD than in CN, whereas one DMR, located in and around *KLF2*, exhibited a higher methylation level in AD (Fig. 4).

**Table 5:** Summary of the validation of six DMRs in two cohorts

					Burden test		Methylation difference^a^
Chromosome	Gene	Amplicon (bp)	Location	Group	*p* value	FDR	(%)
5	*ANKH*	213	intron	2nd cohort	0.0034	0.0447	-4.74
				3rd cohort	0.0633		
				meta-analysis	0.0017		
12	*MARS*	169	exon	2nd cohort	0.0054	0.0467	-5.17
				3rd cohort	0.2452		
				meta-analysis	0.0072		
17	*ANKFY1*	594	intron	2nd cohort	0.0046	0.0467	-5.26
				3rd cohort	0.0680		
				meta-analysis	0.0021		
17	*CHRNE*	615	exon–intron	2nd cohort	0.0013	0.0331	-4.42
				3rd cohort	0.6739		
				meta-analysis	0.0608		
18	*LINC00908*	424	intron	2nd cohort	0.0023	0.0402	-4.53
				3rd cohort	0.0645		
				meta-analysis	0.0011		
19	*KLF2*	536	exon–intron	2nd cohort	0.0006	0.0319	7.37
				3rd cohort	0.4455		
				meta-analysis	0.0015		

^a^For each CpG site in Table S6, the methylation difference in the second cohort was calculated, and the sum of the differences was then averaged

### Validation of 6 DMRs with the third cohort

Methylation changes in the 6 genes were reexamined by BA-seq with the third cohort, composed of 48 CN and 48 AD subjects (none of whom underwent PiB-PET imaging, Table 1). We generated 12 amplicons that covered 108 CpGs in the 6 DMRs from the third cohort, and a bisulfite DNA library for Illumina MiSeq sequencing was again constructed from the 12 amplicons (Table 4). The MiSeq sequencing run resulted in a cluster density of 343 ± 100 (K/mm^2^), a cluster passing filter of 87.09 ± 1.85, and Q30 ≥ 94.2%. The mean read numbers per sample from the CN and AD groups were 29,702 and 32,623, respectively, with average mapping rates of reads from CN and AD being 95.4 and 95.3%, respectively. We did not obtain any reads for an amplicon in the *KLF2* DMR from one AD subject. DNA methylation differences were significant at 96 CpGs among the 108 CpGs surveyed (*P*_*bon*_ ≤ 0.05/108). We also assessed the methylation difference at 6 DMRs in third cohort samples using the Burden test (Table 5). The lowest *p* value was 0.063 at *ANKH,* and the highest was 0.446 at *KLF2*. However, combining data from the second and third cohorts for the 6 DMRs showed a significant methylation difference (*p* < 0.05) in all genes, except for *CHRNE,* which showed a slight difference (*p* = 0.06). We regarded the five DMRs with significant methylation differences as confirmed DMRs.

### Comparison with array-based approaches

**Fig. 5:**
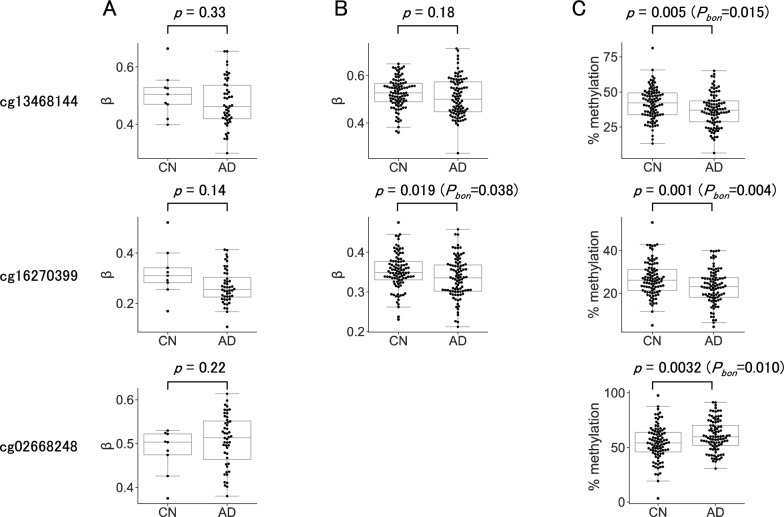
Comparison of methylation levels at CpG sites surveyed by both the array-based method and MC-seq. Array data were downloaded from the public database, Gene Expression Omnibus (GEO), deposited under the accession numbers GSE43414 (A) and GSE144858 (B). The methylation level of each of the blood samples in the array data, shown as a dot, is expressed as a β value, which is derived from the formula: M/(M + U + 100), where M and U are the intensities of fluorescence from the interrogated CpG site with methylated and unmethylated cytosine, respectively. The central bars in the box plots are medians. The significance of the differences between CN and AD groups was examined by Welch’s *t* test. *P*_bon_ is the Bonferroni-corrected *p* value. We were unable to retrieve the cg02668248 data from GSE144858, which may be because it did not pass the quality check

None of the five DMRs we found have been reported in any of the genome-wide methylation studies for identifying AD-related markers in the blood or brain conducted using array-based approaches. To examine the extent to which differences in methylation at CpG sites in DMRs were observed in AD array data, the CpG sites investigated by both methods were extracted from DMRs (Supplementary Table 8). There was only one probe for each of the five DMRs in the currently available EPIC array, and two of the five probes were missed in the formerly distributed HM450 array, showing wider genome coverage by MC-seq than by the arrays. The methylation levels of a few CpG sites in DMRs were extracted from the raw array data of two studies (Fig. 5) [23, 24]. A significant methylation difference was detected at one of the two CpGs in one of these studies. Methylation differences at the remaining CpGs were not significant; however, the directions of their methylation changes in AD were the same as those in the present study when medians were compared.

### Diagnostic ability of AD with DNA methylation

**Fig. 6:**
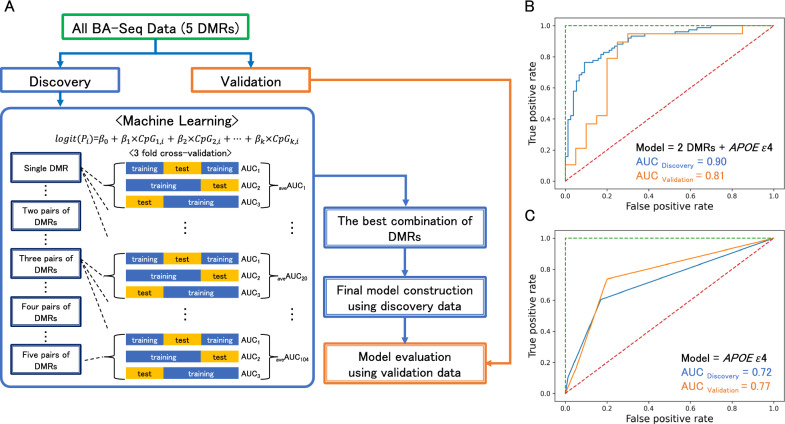
The flowchart for machine learning and receiver operator curves. (A) Flowchart of the machine learning process to discover the best combination of DMRs. ROCs were generated with the combination of *ANKH* and *MARS* methylation and the *APOE* genotype (B) and with only the *APOE* genotype (C) in the discovery (orange) and validation (blue) cohorts

**Fig. 7:**
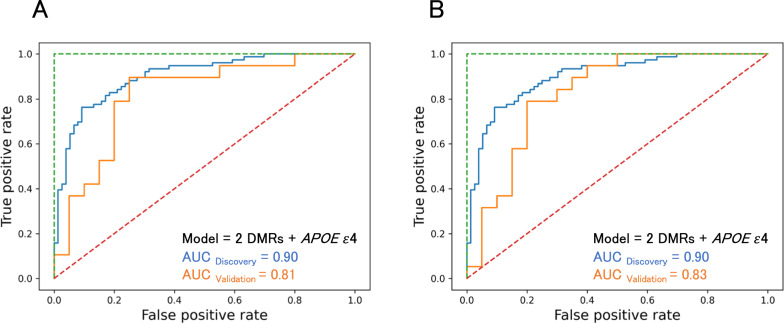
Effect of increased cross-validation for machine learning on diagnostic ability. AUC-ROC analysis incorporating methylation levels of DMRs, adjusting with the *APOE* e4 allele, was performed as in Fig. 6B, but with fivefold (A) and tenfold (B) cross-validations for machine learning. DMRs in *ANKH* and *MARS* were chosen in the diagnostic model of (A), as in Fig. 6B, but *LINC00908* was selected instead of *ANKH* in (B)

We hypothesized that the five DMRs may contain CpG sites capable of discriminating between CN and AD. We selected CpG sites with significant methylation differences between CN and AD (*P*_*bon*_ ≤ 0.05) in the second and third cohort analyses, ensuring that the direction of the methylation change was consistent across both cohorts. The numbers of CpG sites meeting these criteria were as follows: 6 CpGs in *ANKH*, 7 in *MARS*, 14 in *ANKFY1*, 3 in *LINC00908*, and 30 in *KLF2*. The locations of these CpGs are shown in Supplementary Table S6. Methylation levels at these CpGs in the five DMRs, together with the following covariates: age, sex, and the *APOE* ε4 genotype, were used as explanatory variables in a logistic regression analysis to identify the model that best discriminated AD from CN (Fig. 6A). The *APOE* genotype is a well-established risk factor for AD [25]. We performed an AUC-ROC analysis incorporating methylation levels from *ANKH* and *MARS,* adjusting with the *APOE* ε4 allele, which yielded the highest AUC score in the validation set of 0.81 (95% CI: 0.67–0.95, Fig. 6B and Supplementary Table S7). The base model, which used only the *APOE* ε4 genotype, showed a lower AUC of 0.77 (95% CI: 0.69–0.84, Fig. 6C and Supplementary Table S7). Therefore, we concluded that combining methylation levels at specific regions in *ANKH* and *MARS* with the *APOE* genotype provided the best model for diagnosing AD among the combinations assessed. To establish this model by machine learning, we employed a threefold cross-validation. We also examined fivefold and tenfold cross-validations to increase confidence in the threefold model’s performance and ascertain the risk of overfitting. As shown in Fig. 7, similar AUC scores were obtained in both conditions as those in the threefold cross-validation, while a new combination of DMRs was selected in the tenfold cross-validation. These results suggest that the threefold cross-validation was sufficient for machine learning to deduce the diagnostic ability of DNA methylation markers with *APOE* genotypes, and also that different combinations of markers were identified depending on the condition of the cross-validation.

## Discussion

In the present study, we examined the validity of MC-seq in identifying AD-related DMRs in blood for the first time. We obtained 106 DMRs in the first cohort using MC-seq, and 5 DMRs remained after validation in two independent AD and CN cohorts using BA-seq. The low survival rate of DMRs, less than 5%, from the first screening was likely due to the small sample size, which may have led to false positives. A high rate of false positives was theoretically anticipated because the smaller the sample size, the greater the chance of a larger apparent difference in methylation between the two groups of CN and AD due to random variations (statistical fluctuations), even if there was no underlying difference. Therefore, we expected the survival rate to improve with a larger sample size in the first cohort because marker reliability is dependent on sample size [26]. We removed DMRs caused by the sampling bias with two rounds of validation and secured the robustness of the five DMRs that survived and, thereby, that of the results to which the five DMRs were applied. In the second cohort, methylation differences between CN and AD in the five DMRs were approximately 5%, whereas the initial screening criteria required a 15% difference. This suggests that the methylation differences observed in the first cohort were exaggerated due to sample-specific variability as described above. It is also important to note that none of the average methylation differences of the five surviving DMRs met 25%, the stricter threshold we defined. Therefore, the concept of the higher the threshold, the higher the sensitivity for identifying robust markers does not necessarily hold true for limited sample sizes.

We previously detected the same small methylation changes in known AD-associated genes, including *CLU*, *CR1*, and *PICALM* [15]. These findings indicate that DMRs in AD blood may not exhibit large differences, highlighting the need for highly sensitive methods to detect small methylation changes in blood-based biomarkers for AD. The resolution of the current arrays being widely used (e.g., the Illumina HM450 array and EPIC array) currently remains unclear in terms of methylation differences. Therefore, we compared methylation levels at CpG sites measured by MC-seq and methylation arrays using the dataset for AD-related DNA methylation in the blood of Europeans available from the public database. We found a significant methylation change at cg16270399 in one of the two array datasets, and while not significant, the same direction of methylation changes at other CpGs as in MC-seq. These results suggest the availability of array-based methods for finding small methylation changes and the applicability of significant CpGs identified in a Japanese population to other ethnicities. Furthermore, the absence of significant changes in methylation at cg13468144 and cg02668248 in array data may be due to differences in ethnicity among samples, which has been noted [27]. In any case, array-based methods were not expected to detect DMRs because there is only one CpG measurable in each DMR at most on the arrays. MC-seq, which enables the detection of aggregated CpGs with small methylation differences as a DMR, may facilitate the development of novel biomarkers.

It is important to identify preferential regions for DMRs, such as transposable elements (TEs), in order to elucidate the mechanisms by which DMRs are generated. It currently remains unclear whether disease-related DMRs are generated at preferential sites. Since hypomethylation occurs in TEs with aging [28, 29] and carcinogenesis [30], we investigated whether the DMRs detected in the present study were part of TEs, and showed that they were in unique regions using a Blast search (data not shown). The further identification of disease-related DMRs may reveal structural motifs in the genome.

The combination of methylation levels at two of the five DMRs with *APOE* genotypes gave a discriminative power (AUC) of 0.81 for the validation cohort. Due to the small sample size for the first cohort, we expect that increasing the sample size of the first cohort analyzed by MC-seq will result in improved DMRs and, consequently, a higher AUC. Additionally, further improvements may be achieved by using capture probes specifically designed by researchers, as demonstrated in other studies. This approach may enhance the sensitivity and specificity of the method, ultimately improving the identification of reliable biomarkers for AD [31].

We previously showed that AD-related DNA methylation decreased at CpG island shores of *CLU*, *CR1*, and *PICALM* [15]. CpG island shores are located 2 ~ 3 kb from CpG islands and are typically intermediately methylated [32]. In the present study, all 5 DMRs were found in intermediately methylated regions and, except for *KLF2*, showed lower methylation levels in AD. However, these DMRs were too distant from CpG islands to be classified as CpG island shores (Fig. 4). This suggests that, regardless of their positions related to CpG islands, intermediately methylated regions within genes may be susceptible to AD-related methylation changes. Since aging is the most significant risk factor for AD and age-related methylation changes in the human genome [33], it will be interesting to examine whether DNA methylation at these DMRs decreases with age. Hypomethylation in gene bodies has been linked to transcriptional integrity, including spurious transcription initiation [34], mis-splicing [35], and readthrough beyond transcriptional termination signals [36], all of which may decrease the activities of hypomethylated genes. Recent advances in targeted hypomethylation techniques may enable functional analyses of these DMRs in model systems [37]. Two genes identified in this study, *ANKH* and *CHRNE*, have also been genetically related to AD [38, 39]. *ANKH* encodes a transmembrane protein involved in regulating pyrophosphate levels in joints and other tissues. Mutations in *ANKH* may lead to excessive mineralization, contributing to joint pain, arthritis, atherosclerosis, and diabetes. A minor allele of rs112403360, located in an intron of *ANKH*, has been associated with a 1.09-fold increased risk of AD [38]. In contrast, the major allele, considered to be a protective allele against AD, has been associated with cognitively healthy centenarians [40]. Although the mechanism by which the minor allele of the *ANKH* gene increases the risk of AD remains elusive, it may be a hypomorphic allele that affects the metabolism of glucose in the brain because a strong relationship has been reported between genetic variations in *ANKH* and cognitive changes in individuals with normal aging and probable AD using fluorodeoxyglucose-PET [41]. A recent study on a Chinese cohort reported lower methylation levels in an intronic CpG site in individuals with mild cognitive impairment or AD than in cognitively healthy controls [42], and we herein also found lower methylation levels in an intronic region of *ANKH* as a DMR in AD. Hypomethylation in gene bodies has been shown to generate spurious transcription start sites (TSS) [34, 43, 44], which may affect transcription from genuine TSS. Hypomethylation in the *ANKH* gene body may also reduce gene activity by spurious transcription from a hypomethylated region, thereby affecting cognitive function via the hypometabolism of glucose in the brain.

*CHRNE*, which encodes a subunit of the cholinergic receptor, is also genetically associated with AD. A variant in the 3’-UTR region of *CHRNE* has been associated with a 1.09-fold increased risk of AD, possibly by enhancing gene expression [39]. The potential role of *CHRNE* up-regulation in AD is further supported by evidence that the AD-associated SNP rs113260531, located 0.3 Mb upstream of *CHRNE*, has been linked to increased *CHRNE* expression in all four brain regions [45]. Cognitive function may also be affected by a reduction in CHRNE in neurons. Since CHRNE is a component of the receptor for the neurotransmitter, acetylcholine, at synapses, a shortage of CHRNE may lower the sensitivity of neurons to acetylcholine. Further studies are needed to investigate whether hypomethylation of the gene body of *CHRNE* occurs in neuronal cells and reduces the protein due to the generation of spurious TSS.

Another gene harboring a DMR in its coding region, *KLF2*, has also been implicated in AD. *KLF2* belongs to the mammalian KLF family, composed of 17 members (*KFF1*-*KFF17*). All *KLFs* are moderately to be highly expressed in retinal ganglion cells and the brain, playing critical roles in neuronal development, regeneration, and brain homeostasis. Immunohistochemical analyses showed a significant reduction in *KLF2* in AD brains [46]. In mouse models, *KLF2* expression was found to be down-regulated by Ab_1-42_, leading to cerebrovascular dysfunction [47] and hippocampal neuron injury [48], both of which are key pathological features of human AD brains.

**Fig. 8:**
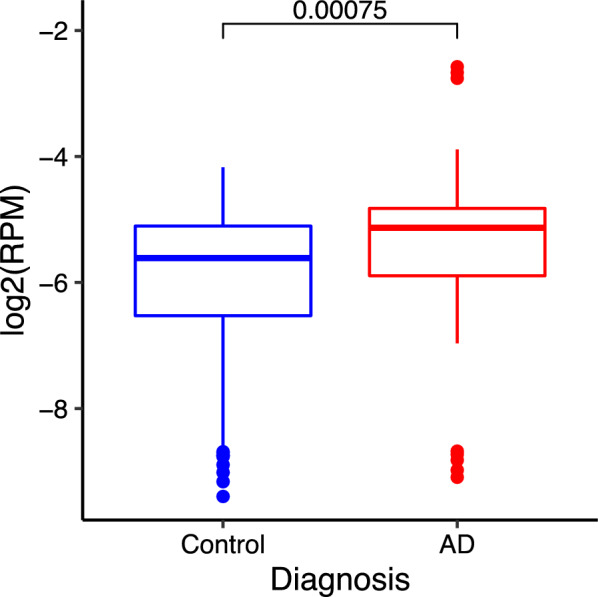
*LINC00908* expression increased in the temporal cortex of AD brains. The *p* value is shown at the top. The image was retrieved from the ADAtlas. https://hanlaboratory.com/AD_atlas/ accessed 18 January 2025. RPM: Reads Per Million

Although limited information is currently available on the functions of the long intergenic non-coding RNA, *LINC00908*, apart from its implications in carcinogenesis, it was recently shown to be up-regulated in the temporal cortex of AD brains (Fig. 8) [49].

Three of the six DMRs identified in blood were located in genes associated with AD. Similarly, we previously detected DNA hypomethylation in three of the six AD-related genes examined in AD blood [15]. This high incidence of methylation changes in AD-related genes in blood suggests that investigating DNA methylation changes in genes may complement genome-wide association studies, particularly for identifying AD-related genes that lack informative DNA variants in or around them.

There are a number of limitations that need to be addressed. The generalizability of the DMRs identified across different ethnicities remains unclear because our analysis was conducted on Japanese blood samples. Furthermore, the timing of DMR emergence in AD patients has yet to be clarified because this study used cross-sectional cohorts. Moreover, we did not perform functional analyses of the genes harboring DMRs. Future research is needed to confirm the methylation changes observed in postmortem human brain tissues because previous studies found only a modest correlation between methylation levels in the brain and blood at most CpG sites [26, 50]. In addition, brain amyloidosis may have been heterogeneous in subjects in the second and third cohorts because biomarkers did not detect it. To estimate the diagnostic ability of DMRs more accurately, they need to be validated in cohorts that are similar to the first cohort, i.e., the PiB-PET-examined cohort. Moreover, the small differences observed in methylation in validated DMRs (~ 5%) may make the practical application of DMRs for diagnostic purposes difficult.

## Conclusions

We employed MC-seq to isolate AD-associated DNA methylation markers for the first time in this study. Despite the small sample size, we identified two methylation markers that efficiently distinguished AD from controls (*ANKH* and *MARS*), achieving a discriminative value AUC of 0.81 when combined with the *APOE* genotype. In future studies, we expect more reliable markers for diagnosing the onset and progression of AD to be identified by applying or modifying the method established in the present study with larger sample sizes and more diverse populations. Furthermore, these markers may be applicable to the stratification of participants in clinical trials for AD that evaluate outcomes because genetic heterogeneity may obscure actual therapeutic effects as expected within genetically unmatched clinical trials [51].

## Supplementary Information

**Additional file 1**.

## Data Availability

Sequence data that support the findings of this study have been deposited in the GEO repository with the accession code GSE24435.

## Abbreviations

AD: Alzheimer’s disease
*ANKH*: *ANKylosis* *Homolog*
APOE: Apolipoprotein E
AUC: Area under the ROC curve
BA-seq: Bisulfite amplicon sequencing
bp: Base pair
*CHRNE*: *Cholinergic Receptor Nicotinic Epsilon Subunit*
CN: Cognitively normal
CpGs: Cytosine–phosphate–guanine dinucleotide sites
DMP: Differentially methylated position
DMR: Differentially methylated region
GRCh37: Genome Reference Consortium Human Build 37
MC-seq: Methylation capture sequencing
MMSE: Mini-Mental State Examination
NCGG: National Center for Geriatrics and Gerontology
NGS: Next-generation sequencing
PiB-PET: Pittsburgh compound-B positron emission tomography
ROC: Receiver operator characteristic
SD: Standard deviation
